# Effect of Cassava Flour Characteristics on Properties of Cassava-Wheat-Maize Composite Bread Types

**DOI:** 10.1155/2013/305407

**Published:** 2013-05-20

**Authors:** Maria Eduardo, Ulf Svanberg, Jorge Oliveira, Lilia Ahrné

**Affiliations:** ^1^Departamento de Engenharia Química, Faculdade de Engenharia, Universidade Eduardo Mondlane, Maputo, Mozambique; ^2^Department of Chemical and Biological Engineering/Food Science, Chalmers University of Technology, SE-41296 Gothenburg, Sweden; ^3^SIK, The Swedish Institute for Food and Biotechnology, SE-402 29 Gothenburg, Sweden; ^4^Department of Process and Chemical Engineering, University College Cork, Cork, Ireland

## Abstract

Replacement of wheat flour by other kinds of flour in bread making is economically important in South East Africa as wheat is mainly an imported commodity. Cassava is widely available in the region, but bread quality is impaired when large amounts of cassava are used in the bread formulation. Effect of differently processed cassavas (sun-dried, roasted and fermented) on composite cassava-wheat-maize bread quality containing cassava levels from 20 to 40% (w/w) was evaluated in combination with high-methylated pectin (HM-pectin) added at levels of 1 to 3% (w/w) according to a full factorial design. Addition of pectin to cassava flour made it possible to bake bread with acceptable bread quality even at concentration as high as 40%. In addition to cassava concentration, the type of cassava flour had the biggest effect on bread quality. With high level of cassava, bread with roasted cassava had a higher volume compared with sun-dried and fermented. The pectin level had a significant effect on improving the volume in high level roasted cassava bread. Crumb firmness similar to wheat bread could be obtained with sun-dried and roasted cassava flours. Roasted cassava bread was the only bread with crust colour similar to wheat bread.

## 1. Introduction

 Bread is an important staple food in South East Africa, providing energy and many nutrients such as proteins, B-vitamins, vitamin E, and minerals. Traditionally in Mozambique, bread is produced by a mixture of wheat flour, yeast (*Saccharomyces cerevisiae*), salt, and water. As wheat is not grown in Mozambique, large quantities are therefore imported at high costs for the country. In order to cut the nation's expenses, the Government of Mozambique has thus mandated the use of composite flour in breadmaking including flour of cassava, maize, or millets that are locally available.

There is a growing interest in using composite flour for breadmaking owing to some economic, social, and health reasons. However, the partial substitution of wheat flour by other flour types presents considerable technological difficulties because their proteins lack the ability to form the necessary gluten network for holding the gas produced during the fermentation [1–3]. The dough formed is more difficult to handle, and the bread has poor loaf volume and crumb softness [4]. Composite flour with cassava has been evaluated in breadmaking, and general observations are reduced loaf volume, crust colour, and impaired sensory qualities as the level of substitution of wheat with other flours increased [5, 6]. Similar results were reported by Khalil et al. [7]; however, acceptable breadmaking potential could be obtained from partial substitution of wheat flour by cassava flour up to 20 and 30% with addition of 1% malt. Recent studies on bread quality from composite cassava-wheat flour have investigated the influence of the baking process, the cassava genotype, and effect of an added hydrocolloid, xanthan gum [8–10]. Very little attention has been paid to investigate the effect of differently processed cassava flour as ingredients in composite flour for breadmaking.

Cassava (*Manihot esculenta *Crantz) is the major food crop produced in Mozambique with maize (*Zea mays* L.) being the second one. A major constraint to cassava utilization is the rapid microbial degradation after harvest. Cassava roots have a shelf life of only 24–48 h after harvest [11]. One way to extend the shelf life of cassava is to prepare a dry product such as flour. In Mozambique three major cassava flour products are prepared for human consumption, sun-dried, fermented, and roasted. Traditionally, cassava flour can be produced from washed or peeled roots, that are grated, chipped, or sliced, then sun-dried on trays, and finally milled into flour [12, 13]. Grated roots may also be pressed to remove excess water and then toasted to produce roasted cassava flour with up to 95% of the starch gelatinized [14]. The grated cassava may also be fermented with lactic acid bacteria, dried, and subsequently roasted in a pan. The resulting flour is called gari and in Mozambique is known as “*rale*.” According to Numfor et al. [15] the fermentation stage improves the internal stability of the starch granules, reduces the swelling power, and decreases the amylose release during heat treatment.

Due to the unique ability of the wheat gluten proteins to form a viscoelastic dough that retains gas during dough leavening [16, 17], wheat flour cannot be substituted directly in a yeast-leavened product without formula modifications [16], for example, by adding bread improvers such as hydrocolloids, enzymes, and emulsifiers. 

Hydrocolloids are used in baked goods primarily to increase moisture retention and to improve viscoelastic properties of the dough in order to improve bread volume. In gluten-free breads based on rice flour and corn starch, Lazaridou et al. [18] showed that addition of hydrocolloids improved dough strength and bread volume. A better specific volume, a softer crumb, and an improved moisture retention were obtained in wheat bread with addition of hydroxypropyl methylcellulose (HPMC) and *κ*-carrageenan [19, 20] and with HM-pectin [21]. Shittu et al. [10] demonstrated improved specific volume and crumb softness in bread made from composite cassava-wheat flour with added xanthan gum. 

The method used to produce the cassava flour (sun-dried, roasted, or fermented) is expected to influence the functional properties of the flour and consequently the bread quality. Information is however scarce regarding the effect of cassava flour type combined with hydrocolloids on bread quality. Therefore, the objective of the present study was to evaluate the quality characteristics of bread baked on composite flour with sun-dried, roasted, and fermented cassava flour, respectively, and with HM-pectin as a baking additive. The proportion of cassava flour in the mixtures varied from 20 to 40% (w/w), and high-methylated pectin was added at levels of 1 to 3% (w/w).

## 2. Materials and Methods

### 2.1. Materials

The ingredients used were wheat flour at 65% extraction rate (Nord Mills), yellow maize flour (AB Risenta, Sweden), granulated white sugar (commercial), refined salt (commercial), instant dry yeast (KronJäst, Jästbolaget AB), margarine (Kondis UM UHF), L(+) ascorbic acid (GR, E. Merck), soy lecithin (Lecico, GmbH), and HM-pectin (DANISCO). All these ingredients were purchased from commercial sources or directly from the suppliers, keeping the same specification in all experiments. About 300 kg of cassava roots (12 months old) was obtained from local producers in Mozambique and then were processed with different methods (sun-dried, roasted, and fermented). One hundred kg of roots were used for each processing method. 

### 2.2. Methods

#### 2.2.1. Processing of Cassava Flour

 Three different traditional processing methods were used to produce flour from cassava roots at the Food Technology Laboratory at University Eduardo Mondlane. For sun-dried flour, the selected cassava roots were washed, peeled, and sliced in small pieces which were sun-dried 1-2 days. For roasted flour, the peeled cassava roots were chipped followed by pressing and screening in a mechanical machine and then toasted in a pan for 10 minutes. For fermented flour, the peeled cassava roots were immersed in water for 5 days that favours growth of lactic acid bacteria [12], and then the fermented roots were crumbled and sun-dried 1-2 days. The dried products per method yield approximately 36 kg, 20 kg, and 25.5 kg, respectively. All dried pieces were ground into flour with a laboratory mill, and excess fibre was removed by passing the ground material through a sieve DIN 4188 (0.125 mm aperture sieve).

#### 2.2.2. Breadmaking Procedure

 Bread dough samples were prepared according to the following formula: 300 g flour (containing different proportions of cassava flour and maize flour, and wheat flour at constant level, see Table 1), HM-pectin at 3, 6, or 9 g levels, 4.8 g yeast, 4.5 g salt, 6.0 g sugar, 9.0 g margarine, 0.3 g ascorbic acid, 1.2 g soy lecithin, and water according to the preceding baking tests. It should be noted that in order to obtain approximately equal consistency of the dough from each of the flour types, the amounts of water added varied as described in Table 1. These limits were set from preliminary experiments, by the quality of the dough being sufficiently good for working into proper bread. All the ingredients were mixed in a kitchen mixer KS90 (KitchenAid, USA) for 2 min at low speed (speed setting: 1) and for 8 min at medium speed (speed setting: 2), until the dough was well developed. The temperature of the dough was about 22°C. After mixing, the dough was covered with a kitchen cloth and left to ferment at room temperature for 45 min. After the first fermentation, the dough was divided into 50 g portions, rounded, placed into bread pans, and proofed for another 45 min in a fermentation cabinet (LabRum Klimat AB, Stockholm, Sweden) at 30°C and 80% relative humidity. The proofed dough was baked at 220°C for 7 min in an oven (Therma Grossküchen, *Le Chef*, Sweden) with air circulation. Ten miniloaves were thus produced in each batch. Then, the bread was cooled for 60 min at ambient temperature. A control wheat bread was prepared simultaneously in the same oven under identical conditions to those of the experimental design. All measurements were taken using one batch as one collective unit.

#### 2.2.3. Experimental Design

 The baking experiments were planned according to a full factorial design with 3 levels for types of cassava flour (sun-dried, roasted, and fermented) with 2 levels for amounts of cassava and HM-pectin, plus a center point (for sun-dried pretreated cassava flour only), replicated three times. This resulted in 15 experiments that were performed in random order. The design is shown in Table 1. It is noted that the amount of wheat flour was always 150 g, while that of maize + cassava flour was also always equal to 150 g, so the only control factor regarding flour composition is the ratio cassava-to-maize. The second control factor is the amount of HM-pectin (3, 6 or 9 g) while the third is the type of pretreatment. Water is actually a noise factor (source of variability and experimental error), which must vary as shown in Table 1 so that the consistency of doughs prepared would be similar. 

Six miniloaves were analysed for each response in each batch, totalling 15 × 6 = 90 data points for each response.

#### 2.2.4. Moisture Content

 The moisture content of the flour samples was measured by weight difference before and after drying of the samples in a vacuum oven [22]. All flour samples were analyzed in triplicate.

#### 2.2.5. Water Absorption of Flour

The water absorption of the flour samples was determined by the method modified by Anderson et al. [23]. Five gram of each sample was weighed into a centrifuged tube, and 30 mL of distilled water was added and vigorously mixed. The samples were allowed to stand for 30 min and centrifuged (Beckman GP, UK) at 3,000 rpm for 15 min. The top layer was decanted off and the sample weighed again. The amount of water in the sample was recorded as weight gain (g/g flour) and was taken as water absorbed. 

#### 2.2.6. Microstructure of Starch Granules

The starch samples obtained from sun-dried, roasted, or fermented cassava roots were prepared using the smear method as reported by Hongsprabhas et al. [24] and examined with bright-field (BFM) and polarized light microscopy (PLM). The water slurries of cassava flour (soaked overnight at room temperature) were smeared onto an object glass. After drying, the samples were stained with Lugol's iodine solution and covered with a glass sealed with nail polish. The samples were thereafter examined with a Microphot FXA light microscope (Nikon, Tokyo, Japan) using a 10x and a 40x magnification. Images were taken with an Altra 20 Soft Imaging System camera (Olympus, Tokyo, Japan).

#### 2.2.7. Bread Volume

The loaf volume was measured one hour after the end of the baking process by the displacement method in which alfalfa seed was used instead of millet. The average of six measurements was recorded as the loaf volume (cm^3^).

#### 2.2.8. Crumb Structure

The bread crumb structure was evaluated from images captured using a flatbed scanner (MiraScan, BenQ, Version 5.10). Images were scanned with full scale at 100 dots per inch.

#### 2.2.9. Crust Colour

The crust colour was measured on the bread surface using a Colour Reader CR-10 (Konica Minolta Sensing, Japan). *L* ^*^ *a* ^*^ *b* ^*^ values were recorded and the results reported as brownness index (BI), calculated according to Maskan [25]

(1) $$\begin{array}{c} \text{BI}=\frac{\left[100\cdot \left(x-0.31\right)\right]}{0.17}, \end{array}$$

where *x* is defined as

(2) $$\begin{array}{c} x=\frac{a+1.75L}{5.645L+a-3.01b}, \end{array}$$

where *a* ^*^ is redness, *b* ^*^ is yellowness, and *L* ^*^ is lightness. 

The average of four measurements was taken as the crust colour parameter. 

#### 2.2.10. Crumb Firmness

Slices of each loaf (2.5 cm) were taken for analysis of the crumb firmness, measured 6 h after baking using an Instron Universal Testing Machine (UTM, model 5542). A modified AACC standard method 74-09 was used with a cylindrical probe (diameter 15 mm). The probe compressed the slices 40% at a test speed of 1.7 mm/s. The compression curves of the bread crumbs were recorded automatically by the BlueHill software (Merlin, version 5, Instron Corp., Canton, MA, USA). The Young modulus readings between 3 and 20% were taken as a measure of bread crumb firmness. The measurements were carried out on 15 mm thickness slice taken from the centre of the bread loaf. The measurements were carried out on four loaves from each batch. 

#### 2.2.11. Statistical Analysis

A factorial ANOVA was applied to the full factorial design only (i.e., without using the data obtained for sun-dried cassava with middle settings for cassava level and pectin content), to quantify the relative significance of each of the control factors and all two-way interactions between factors, using Statistica software (v.8, StatSoft Inc.). 

The data were also subjected to a least squares regression analysis with a multifactorial model using the type of cassava flour pretreatment as a dummy variable using Statistica (v8, StatSoft Inc.). The model is

(3) Y=ys+csC+psP+isCP+qs(1−Df−Dr)C2  +yfDf+cfDfC+pfDfP+ifDfCP+yrDr+crDrC+prDrP+irDrCP,

where *Y* represents any of the responses measured (bread volume, firmness, and colour) and *C* and *P* are the coded values of the factors cassava level and pectin content, respectively (0, 0.5, or 1, as per Table 1). The model uses sun-dried bread with low cassava and pectin content as a reference, so *y*_*s*_ is the average model value for this bread. *D*_*f*_ is the dummy variable indicating whether the pretreatment used was fermentation ( = 1 if yes, 0 otherwise), and *D*_*r*_ is the similar dummy variable indicating roasted pretreatment ( = 1 if yes, 0 otherwise). 

As the model is additive, it is straightforward to obtain the estimated value for any type of bread by simply adding the respective parameters. The results of the regression analysis are presented in modified Pareto charts.

## 3. Results and Discussion

### 3.1. Volume

Volume is an important quality characteristic of bread, and that is negatively affected when wheat is replaced by cassava. The results of the factorial ANOVA for bread volume are shown in Table 2. The cassava level was the factor that had the largest effect, 27%. The type of pretreatment and pectin content had less influence, while the interactive effect between cassava level and pectin content was also small. However, the interactive effects of pretreatment with cassava level and with pectin content were larger. 

**Figure 1:**
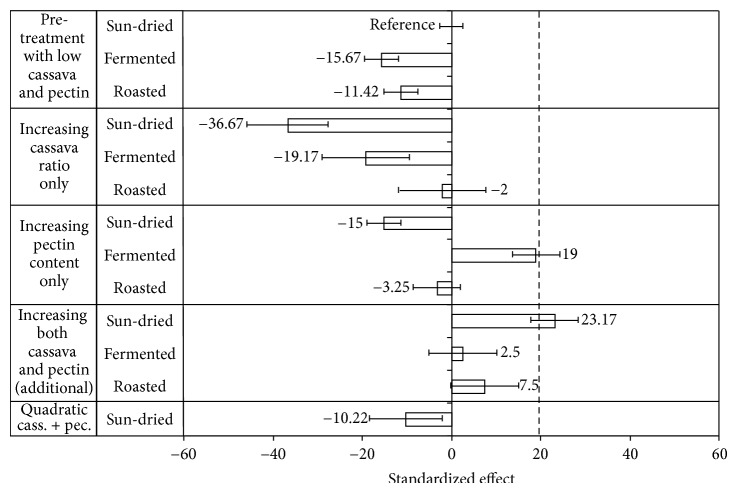
Modified Pareto chart showing the relative effects of pretreatments, flour composition factors, and the interaction of these on bread volume according to the regression model (*R* ^2^ = 0.75). Bread made with sun-dried flour with low cassava and low HM-pectin contents was set as reference bread. The volume of wheat bread is indicated by a dotted line. Effects with error bars not including point 0 are statistically significant at 95% confidence level.

The results of the regression model are shown in Figure 1. The results show that for roasted cassava a change in level or pectin content had no effect on bread volume. For the other types of cassava (fermented or sun-dried), increasing the cassava level clearly caused a volume decrease. All types of bread with cassava flour were smaller than the wheat flour control bread that had a volume of 135.0 cm^3^; however the differences were considered acceptable. The largest bread volume of all cassava bread was obtained with fermented cassava (20%) and high pectin content, 118.3 cm^3^ (115.0 − 15.67 + 19.00, see Figure 1). Increasing the fermented cassava level would negate the increase achieved with higher pectin (effects around +19 and −19, with negligible interactive effect).

In the baking experiments with the full factorial design the amount of water added in the dough preparations ranged from 230 to 236 g with sun-dried and fermented cassava flour and from 236 to 265 g with roasted cassava flour. The amount added was determined by taking in account the handling properties of the doughs. If the same amount of water was added to all the doughs, it would have been impossible to form bread from the dough. To evaluate the effect of water on bread volume, the amount of water was increased in doughs prepared with sun-dried and fermented cassava flour, to obtained similar amount of water as the one used for roasted. The addition of 255 g of water resulted in a very sticky dough but still workable, and slightly increased (not statistically significant) volume was observed.

Increasing the cassava ratio decreased the volume in the bread made with sun-dried and fermented flour which is quite evident from the results shown in Table 3. However, the cassava content had no effect on volume of roasted cassava bread, due to the interaction with pectin content, which once again was particularly dramatic for this pretreated flour. While increasing the cassava ratio with the lower pectin content had no effect on the bread volume, it resulted in a slightly increased bread volume with the higher pectin content. 

 The overall effect of a reduced bread volume with cassava flour in the flour mixture can be explained by reduced flour strength and a lower ability of the gluten network to enclose the carbon dioxide produced during fermentation. Similar findings on loaf volume of composite flours were reported by other researchers [1, 5–7]. In baked products, hydrocolloids influence the dough rheology and bread quality parameters. An increase in volume of composite bread formulations due to pectin addition may be attributed to improvement of dough development and gas retention by increasing dough viscosity [19, 26] and dough stability [27]. HM-pectin does contain hydrophobic groups that might induce interfacial activity with gluten and thereby forming gel networks during the breadmaking process. HM-pectin has also been shown to interact with wheat starch causing an increased paste viscosity during heating [28]. Both the increased viscosity and the gel network might strengthen the gas-holding properties of the expanding cells in the dough and consequently result in a higher loaf volume [29]. Lazaridou et al. [18] reported that pectin at 2% level was the only hydrocolloid of five tested (CMC, xanthan, Agarose, oat *β*-glucan) that increased the volume of gluten-free breads. High-methylated pectin has also shown to both increase dough volume and the specific volume of wheat bread [21]. The roasted cassava provides a soft agglomerate of gelatinized starch granules [14] that could provide an additional built-up of an interlinked network with the HM-pectin in the dough explaining the increased volume of the bread with roasted cassava compared with the other two cassava flour types.

The higher bread volume with fermented cassava and high pectin content could be related to the prereatment conditions given to the cassava flour. The fermentation process of cassava which took place during 5 days at ambient temperature and subsequent sun-drying has previously shown to result in better baking expansion during dough preparation [30]. Furthermore, these authors pointed out that the baking expansion will increase with starch disintegration and degradation during cooking. The influence of pectin on bread volume with fermented cassava addition might be explained by the increase in paste viscosity that slows down the rate of gas diffusion and allows its retention during the early stage of baking. Mestres et al. [30] found that the expansion ability of fermented cassava was negatively correlated with paste viscosity.

### 3.2. Firmness

Bread volume has a direct influence on the firmness of bread; however firmness is also influenced by the strength of the crumb formed. The results of the factorial ANOVA for bread firmness are shown in Table 2, and the most influential factor on bread firmness is the type of pretreatment applied to cassava flour explaining 40% of the effect, followed by its interactive effects. In particular, interactive effects of pretreatment with cassava ratio and pectin content vary for the differently pretreated cassava flour types.

**Figure 2:**
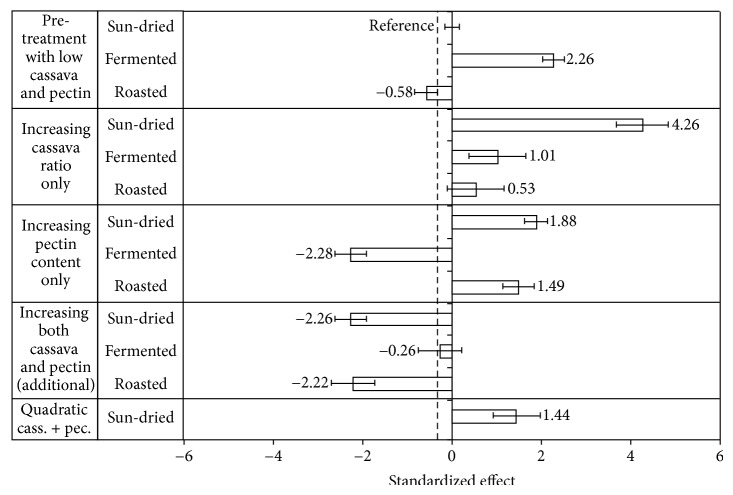
Modified Pareto chart showing the relative effects of pretreatments, flour composition factors, and the interaction of these on bread firmness (N) according to the regression model (*R* ^2^ = 0.95). Bread made with sun-dried flour with low cassava and low HM-pectin contents was set as reference bread. The firmness of wheat bread is indicated by a dotted line. Effects with error bars not including point 0 are statistically significant at 95% confidence level.

Figure 2 shows more detailed results in a modified Pareto chart. The effects are related to the reference bread which was made with low level (20%) of sun-dried cassava and low pectin content, with a firmness of 7.66 ± 0.17 N. Bread with fermented cassava flour results in firmer bread crumb when cassava level and pectin content were both low, and softer bread types were obtained with roasted flour (*P* < 0.05). However, the bread types made with roasted cassava and sun-dried cassava both had a firmness that was similar to that of the control wheat bread, 7.33 ±0.50 N (dotted line). Increasing the cassava level caused substantially higher firmness of the bread made with sun-dried cassava with an increase in firmness of 4.26 N. Increasing the pectin content had a softening effect in breads made with fermented flour compared with bread types of the other two cassava flour types. The effect of increasing both cassava ratio and pectin content, however, showed a significant interactive effect when using sun-dried or roasted cassava but not fermented. 

Table 3 shows that the effect of the pretreatment is significant at higher cassava levels, with sun-dried flour giving firmer bread and roasted flour softer bread. The nature of the quadratic effect is also visible in a slight curvature of the effect of increasing the ratio of sun-dried cassava flour.  Table 4 shows that the pectin content had very little effect in flours made with sun-dried or roasted cassava, but increasing its content with fermented cassava resulted in bread that were softer, becoming similar in firmness to those made with roasted flour at the higher pectin content. Finally, the interactive effect between cassava level and pectin content showed that increasing the cassava level increased the firmness in all bread, except in the ones with roasted cassava and high pectin content (Table 4).

These results show that substitution of wheat flour with increasing amounts of cassava flour will affect the bread crumb firmness differently depending on the pretreatment of the cassava flour. A necessary property of the starch component is to support the elastic strength of the diluted gluten network responsible for the gas-holding capacity of the dough. Evidently, the starch properties of the three different cassava flour types interact differently with the protein-gluten network during the baking process resulting in different effects on the crumb structure and thus the firmness.

The softening effect of the roasted cassava flour might be attributed to pregelatinization of the cassava starch during roasting (heat-moisture treatment), providing a high swelling capacity at the dough phase. Furthermore, pregelatinized cassava starch granules remain resistant against disintegration at higher temperatures, up to 90°C, and form soft agglomerates [14] that might contribute to a softer bread crumb structure during baking off. 

Numfor et al. [31] observed that the average starch granule diameter, solubility, and swelling power were found to be depressed by fermentation. Formation of amylose-like fragments was suggested to interact with the starch granules thus resulting in greater internal granule stability. A more stable granule would account for the observed reduced solubility and swelling power of the fermented starches. This, in turn, would account for a reduced ability to support an elastic network during baking off and result in firmer bread crumb. However, adding pectin to this structure compensates for this effect, resulting in bread similar to the wheat bread control when using the higher cassava ratio.

 The softening effect on bread crumb at high levels of HM-pectin (3%) that was obtained with fermented or roasted cassava might be explained by the interaction of the hydrocolloid with the amylose gel formation. Lower viscoelastic properties have been demonstrated in gels of amylose/hydrocolloid mixtures [32]. Furthermore, hydrocolloids have been shown to retard the retrogradation of cassava starch gels [33], and HM-pectin at 1% level resulted in a reduced crumb firmness of wheat bread [21].

### 3.3. Brownness

The colour of the bread is important for consumers, and it is related with the extension of the Maillard reactions. The results of the factorial ANOVA for brownness are shown in Table 2. Brownness is significantly influenced by the type of pretreatment which explains 85% of the effect, with pectin content and cassava level having a small influence.

**Figure 3:**
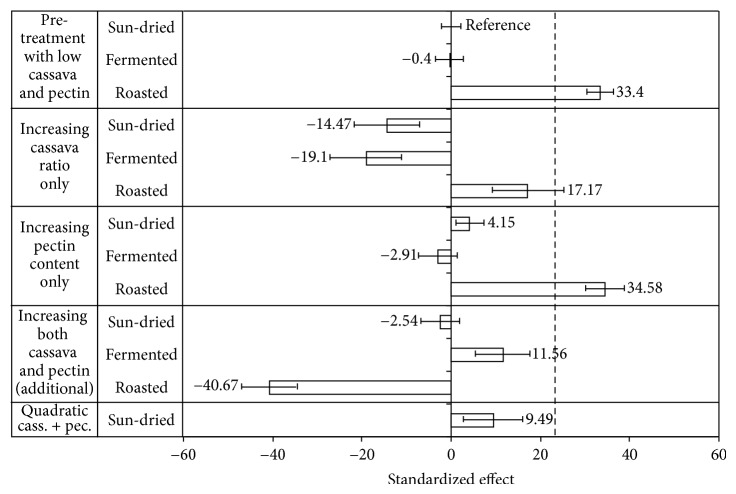
Modified Pareto chart showing the relative effects of pretreatments, flour composition factors, and the interaction of these on brownness according to the regression model (*R* ^2^ = 0.93). Bread made with sun-dried flour with low cassava and low HM-pectin contents was set as reference bread. The brownness index of wheat bread is indicated by a dotted line. Effects with error bars not including point 0 are statistically significant at 95% confidence level.

It is evident from the model results shown in Figure 3 that roasting causes a significant increase in brownness, and this was the only treatment able to provide bread with a brown colour similar to that of the control wheat bread. The other pretreatments resulted in breads with a brownness index value of about 58 that was significantly lower than the value measured in wheat bread, 91.8 ± 2.3. Increasing the pectin content makes the bread made from roasted cassava even browner, although this effect has a strong interaction with increasing the cassava level. Increasing the pectin content seems to have little effect on the other bread types, while increasing the cassava ratio makes them less brown. This effect was also demonstrated by increasing the roasted cassava ratio in bread that became less brown with high pectin content, but with low pectin content the brownness increased substantially (*P* < 0.05), Table 4.

The effect of browning of the composite bread types can be attributed to Maillard reactions between wheat proteins and reducing sugar [34]. The Maillard reactions are also related to temperature, time, and the presence of water (moisture), and in bread crust the temperature and water activity might be optimal for browning reactions [35].

In bread with roasted cassava the pregelatinized starch released some amylose units that expose reducing glucose ends that can participate in browning reactions [14]. With high levels of pectin the amount of available water in the dough might be reduced resulting in a lower rate of browning. 

### 3.4. Crumb Structure

**Figure 4:**
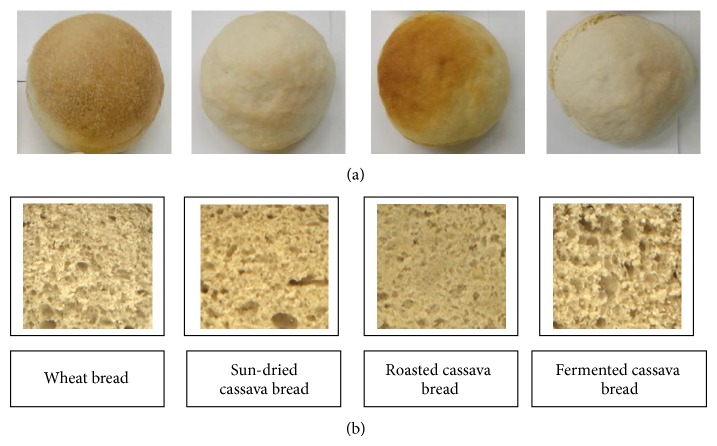
Composite bread made with 40% cassava flour and 3% of HM-pectin. Crust colour (a) and crumb structure (b).

Pictures of breads baked with 40% cassava and 3% HM-pectin (Figure 4) show the crust colour and crumb structure. The crumb structure of the breads prepared with roasted or sun-dried cassava flour was characterized by rather small and uniformly distributed pores with some few big pores of irregular shape. The crumb structures of these breads were similar to the wheat bread. However, an expandable crumb structure was noticed with fermented cassava flour in the bread. Compared to the other two cassava types, the bread baked with roasted flour had significantly better crust colour and similar to the wheat bread.

### 3.5. Cassava Starch Granules Microstructure

In order to understand the different bread characteristics obtained with the different cassava flour types, the microstructure of the cassava starch granules was analysed by bright-field (BF) and polarized light microscopy (PLM). 

**Figure 5:**
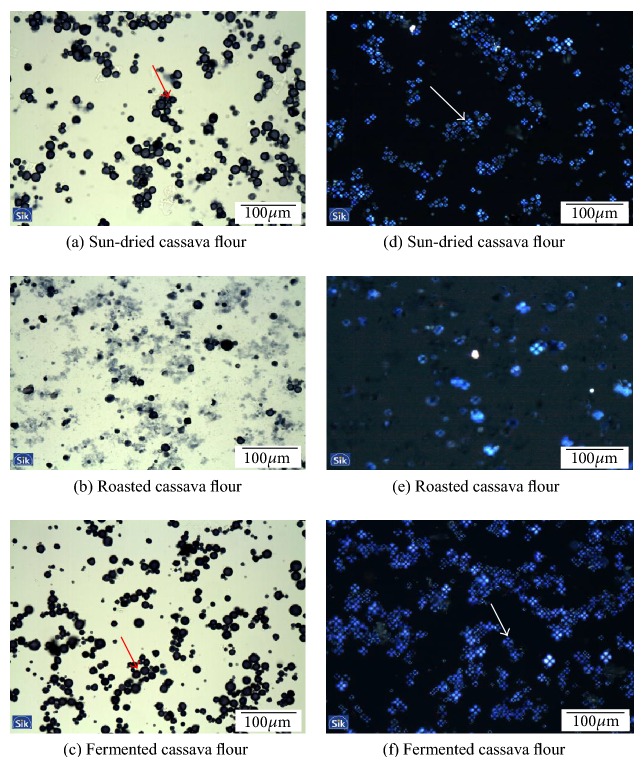
Bright-field (a, b, and c) and polarized Light (d, e, and f) micrographs of starch granules. Sun-dried and fermented cassava flour with intact granules ((a) and (c), red arrows) and roasted cassava flour with partly swollen granules, few Maltese crosses (e) compared with (d) and (f).

Micrographs of the cassava granules suggest that there is a structural difference among the three types of cassava. In roasted cassava flour, partially swollen starch granules are observed, whereas in others two intact granules are a dominating feature (Figures 5(a), 5(b), and 5(c)). The partially swollen starch granules resulted in significantly higher water absorption, 236% in the roasted cassava flour compared to 102% in the fermented cassava flour and 136% in the sun-dried cassava flour. It thus appears that starch characteristics are important factors that will influence water absorption in the three flour types of cassava. The water absorption data reflects that a larger amount of water is needed in the preparation of doughs with roasted cassava flour to obtain similar dough characteristics as with the other cassava flours. Similar findings have been reported by Defloor et al. [6].

Polarized light microscopy of sun-dried and fermented cassava starch granules showed the typical Maltese cross phenomenon (Figures 5(d), 5(e), and 5(f)) indicating a large number of nongelatinized starch granules. Mestres and Rouau [36] have earlier shown that fermentation and drying of cassava do not change the gelatinization properties of the starch granules.

## 4. Conclusion

The type of processed cassava flour has large effect on the quality parameters of cassava-wheat-maize composite breads. With high level of cassava, bread with roasted cassava flour had a significantly higher volume compared with bread made of sun-dried or fermented cassava flour. The pectin level had a significant effect on improving the volume in high level roasted cassava bread. Crumb firmness as well as crumb structure of sun-dried bread and of roasted cassava bread was similar to wheat bread. Composite bread types with roasted cassava were the only bread types with crust colour similar to wheat bread.

In relation to the important objective of achieving bread similar to that made with wheat flour, in terms of volume and firmness, roasted cassava flour is the most promising pretreatment. 

In terms of sensory properties, a preliminary consumer acceptance test performed in Maputo, Mozambique, indicated that composite wheat-maize-cassava flour (sun-dried/roasted) bread had an overall acceptability similar to wheat bread. A more comprehensive consumer acceptance study is underway to be performed.

## Figures and Tables

**Table 1:** Dough formulae of bread samples in the experimental design.

Run order	Composition of the dough					Pretreat∗
	Water (g)	Flour types(g)			Pectin (g)	
		Wheat	Maize	Cassava		
13	230	150	90	60	3	S
5	234	150	30	120	3	S
14	233	150	90	60	9	S
11	236	150	30	120	9	S
6	236	150	90	60	3	R
10	254	150	30	120	3	R
4	236	150	90	60	9	R
8	265	150	30	120	9	R
7	230	150	90	60	3	F
15	233	150	30	120	3	F
1	232	150	90	60	9	F
3	235	150	30	120	9	F
12	232	150	60	90	6	S
2	232	150	60	90	6	S
9	232	150	60	90	6	S

^*^Pretreatment of the cassava flour. S: sun-dried; R: roasted; F: fermented.

**Table 2:** Direct and interaction effect of bread quality parameters.

Factor/interaction	%		
	Volume	Firmness	Brownness
Main effects			
Pretreatment (Pt)	6∗	40∗	85∗
Cassava ratio (Cr)	27∗	11∗	3∗
Pectin content (Pc)	5∗	2∗	1∗
Interactions			
Pt × Cr	18∗	20∗	1
Pt × Pc	15∗	17∗	1
Cr × Pc	4∗	5∗	1∗
Error	25	5	7

SS_total_	12664	212.5	59418

^*^Effects are statistically significant at a 90% confidence level.

**Table 3:** Mean values of firmness (N) and volume (cm^3^) for bread types made with sun-dried, roasted, and fermented cassava. The means for the different cassava ratios with the standard error being due to white noise and to the influence of pectin content.

Cassava type	Firmness (N)	Volume (cm^3^)
Sun-dried		
20%	8.6 ± 0.4	107.5 ± 4.1
30%	11.0 ± 0.3	90.5 ± 2.9
40%	11.7 ± 0.2	82.4 ± 3.4
Roasted		
20%	7.8 ± 0.3	102.0 ± 2.2
40%	7.3 ± 0.2	103.7 ± 2.4
Fermented		
20%	8.8 ± 0.5	108.8 ± 4.3
40%	9.7 ± 0.6	90.9 ± 5.4

**Table 4:** Mean values of firmness (N), brownness index (BI), and volume (cm^3^) of bread types made with roasted cassava and containing 1 or 3% pectin content.

Cassava type	Firmness		Brownness		Volume	
	Pectin content					
	1%	3%	1%	3%	1%	3%
Roasted						
20%	7.1 ± 0.1	8.6 ± 0.1	91.8 ± 0.2	126.4 ± 2.2	103.6 ± 5.0	100.3 ± 4.4
40%	7.6 ± 0.2	6.9 ± 0.4	109.0 ± 5.3	103.3 ± 4.7	101.6 ± 5.5	105.8 ± 3.8

Sun-dried	9.8 ± 0.9	10.5 ± 0.4				
Roasted	7.4 ± 0.1	7.7 ± 0.4				
Fermented	10.4 ± 0.3	8.0 ± 0.2				
